# Investigating of the role of CT scan for cancer patients during the first wave of COVID-19 pandemic

**DOI:** 10.1016/j.redii.2022.100004

**Published:** 2022-03-31

**Authors:** Sylvain Bourdoncle, Thomas Eche, Jeremy McGale, Kevin Yiu, Ephraïm Partouche, Randy Yeh, Samy Ammari, Hervé Rousseau, Laurent Dercle, Fatima-Zohra Mokrane

**Affiliations:** a Radiology Department, Rangueil University Hospital, 1 avenue du Professeur Jean, Poulhes, 31059, Toulouse France; b Columbia University Vagellos College of Physicians and Surgeons, Department of Radiology, New York, New York City, USA. Department of Radiology New York Presbyterian Hospital, United States; c Memorial Sloan Kettering Cancer Center, Molecular Imaging and Therapy Service. New York, United States; d Département de l'Imagerie Médicale, Service d'Imagerie Diagnostique, Gustave Roussy, Université Paris Saclay, Villejuif, France; e BIOMAPS. UMR1281. INSERM.CEA.CNRS.Université Paris-Saclay, France; f Montefiore Medical Center, Albert Einstein College of Medicine, Bronx, NY, United States

**Keywords:** COVID-19, Multidetector computed tomography, Meta-analysis, Medical oncology

## Abstract

**Introduction:**

Amidst this current COVID-19 pandemic, we undertook this systematic review to determine the role of medical imaging, with a special emphasis on computed tomography (CT), on guiding the care and management of oncologic patients.

**Material and Methods:**

Study selection focused on articles from 01/02/2020 to 04/23/2020. After removal of irrelevant articles, all systematic or non-systematic reviews, comments, correspondence, editorials, guidelines and meta-analysis and case reports with less than 5 patients were also excluded. Full-text articles of eligible publications were reviewed to select all imaging-based publications, and the existence or not of an oncologic population was reported for each publication. Two independent reviewers collected the following information: ( 1) General publication data; (2) Study design characteristics; (3) Demographic, clinical and pathological variables with percentage of cancer patients if available; (4) Imaging performances. The sensitivity and specificity of chest CT (C-CT) were pooled separately using a random-effects model. The positive predictive value (PPV) and negative predictive value (NPV) of C-CT as a test was estimated for a wide range of disease prevalence rates.

**Results:**

A total of 106 publications were fully reviewed. Among them, 96 were identified to have extractable data for a two-by-two contingency table for CT performance. At the end, 53 studies (including 6 that used two different populations) were included in diagnosis accuracy analysis (*N* = 59). We identified 53 studies totaling 11,352 patients for whom the sensitivity (95CI) was 0.886 (0.880; 0.894), while specificity remained low: in 93% of cases (55/59), specificity was ≤ 0.5. Among all the 106 reviewed studies, only 7 studies included oncologic patients and were included in the final analysis for C-CT performances. The percentage of patients with cancer in these studies was 0.3% (34/11352 patients), lower than the global prevalence of cancer. Among all these studies, only 1 (0.9%, 1/106) reported performance specifically in a cohort of cancer patients, but it however only reported true positives.

**Discussion:**

There is a concerning lack of COVID-19 studies involving oncologic patients, showing there is a real need for further investigation and evaluation of the performance of the different medical imaging modalities in this specific patient population.

## 1 Introduction

The COVID-19 pandemic has dramatically changed medical, economic, and social practices worldwide. In early 2020, the virus spread quickly around the globe, rapidly contributing to the largest pandemic outbreak since the great influenza crises of 1918 and 2009 [1]. This pandemic has had a disproportionate impact on healthcare systems, both directly by overloading hospitals with COVID patients and indirectly by modifying how unaffected patients—those without an active COVID infection—are managed. While these consequences have been sparsely investigated in pediatric [2] and pregnant [3] populations, there is an overall paucity of research in this area and many studies are burdened by limitations such as the use of purely retrospective patient cohorts. Moreover, there are even fewer studies evaluating the lasting effects of the pandemic on cancer patients specifically [4], despite the fact that cancer remains a leading cause of death worldwide. As cancer treatments continue to improve, and the population of “chronic” oncologic patients grows, it will be of utmost importance to improve detection of emergent diseases in the setting of pre-existing cancer.

With COVID-19, as with many diseases of the 21st century, there exists a central role for imaging in diagnosis, management, and prognostication. Chest Computed Tomography (CT), in particular, has demonstrated potential clinical utility due to its accessibility, speed, and relatively high diagnostic sensitivity for COVID-19 detection, ranging from 61% [5] to 99% [6]. Specificity is also promising, though a bit more controversial, with recent studies indicating performances up to 91% [7], dramatically improved from older publications hovering around 25% [8]. It is important to note that these metrics are achieved in the absence of underlying disease, in patients with a presumed “normal” pre-infection CT appearance of pulmonary parenchyma. In theory, pre-existing parenchymal abnormalities, like those widely observed in cancer patients, or related drug-induced pulmonary changes, may compromise the performance of chest CT in diagnosing active COVID-19 infection. However, how exactly this underlying disease impacts imaging performance is yet to be fully explored, and thus the applicability of diagnostic chest CT remains disputed [9, 10] and seen less favorably than the gold standard of RT-PCR. The main objective of the present study, conducted during the first wave of the COVID-19 pandemic, is to perform a systematic review and meta-analysis comparing the performance of chest CT for the diagnosis of COVID-19 in patient populations with and without cancer.

## 2 Materials and methods

### 2.1 Study selection and literature search strategy

The study protocol was developed and previously registered in PROSPERO with the following registration number CRD42020184819.

For our purposes, a systematic search of the major reference database MEDLINE (PubMed), was undertaken in April 2020. Two major imaging databases built for the COVID-19 pandemic, Radiology [11] and European Radiology [12], were also included in the search. The study was conducted according to the Preferred Reporting Items for Systematic Reviews and Meta-Analyses (PRISMA) guidelines. Key search terms included “CT" AND “COVID-19". Details of search terms used for each database are reported in Table 1. All articles from 01/02/2020 to 04/23/2020 were screened. After removal of duplicate articles and publications including only an abstract, we automatically excluded reports that were not in English or that were non-human studies, case reports with less than 5 patients, systematic or non-systematic reviews, comments, correspondences, editorials, guidelines, and meta-analyses. The commercial bibliographic management software used was EndNote X9.3.1.

**Table 1:** Search Strategy for MEDLINE (Pubmed).

Keywords	("CT findings" OR "CT scan" OR "CT-scan") AND ("COVID-19"OR "COVID19″ OR "COVID 19")
Publication period	01/02/20 – 04/23/20

### 2.2 Inclusion and exclusion criteria

Titles and abstracts of articles were initially screened to select eligible publications, and we removed those with the following characteristics: (1) Publications with data other than imaging; (2) Abstracts with unavailable full-text versions; (3) Studies investigating the diagnostic value of ultrasound (US), chest X-ray or other imaging modalities; (4) Studies focused on pregnant women or newborns.

All studies identified by the search were screened for eligibility by two independent authors (S.B and F.Z.M) blinded to each other's decisions. In case of disagreement, a consensus was reached by a third reviewer (E.P)

### 2.3 Data extraction

Two reviewers (S.B and F.Z.M) extracted the following data from each selected imaging-based article: (1) Authors, journal and year of publication, country of origin; (2) Study design characteristics; (3) Demographics as well as clinical and pathological variables with percentage of cancer patients if available; (4) Imaging performance metrics such as sensitivity, specificity, and contingency tables. Table 2 summarizes all extracted data. The two investigators (S.B, F.Z.M) assessed all studies independently. Disputes were discussed with a third reviewer (L.D) and resolved by consensus.

**Table 2:** Extracted relevant data.

General publication data	Title Authors Journal Date of publication Country
Study design and characteristics	Retrospective study Prospective study Case report / ncase series Editorial Consensus conference Correspondence / comment Review / Meta-analysis Monocentric National multicentric International multicentric
Population	Percentage of oncologic patients Specific population (pediatric – pregnant)
Diagnostic performance	Sensitivity Specificity TP/FP/FN/TN

Abbreviations: CT:computed tomography, TP: true positives, FP: false positives, FN: false negatives, TN: true negatives.

### 2.4 Data analysis

The sensitivity and specificity of chest CT were pooled separately using a random-effects model. The positive predictive value (PPV) and negative predictive value (NPV) of CT as a diagnostic test was estimated for a wide range of disease prevalence rates. Studies without extractable data were excluded from the meta-regression analysis. Sensitivity analysis was conducted for chest CT. Additionally, all duplicate studies were removed.

### 2.5 Statistical analysis

Analyses were conducted using Microsoft Excel (v2019, Microsoft, USA, 2019) and open-source R software (version 3.6.2; R Foundation for Statistical Computing, Vienna, Austria). A p-value less than 0.05 was considered indicative of statistical significance (α = 0.05).

## 3 Results

### 3.1 Identification and selection of studies

**Fig. 1:**
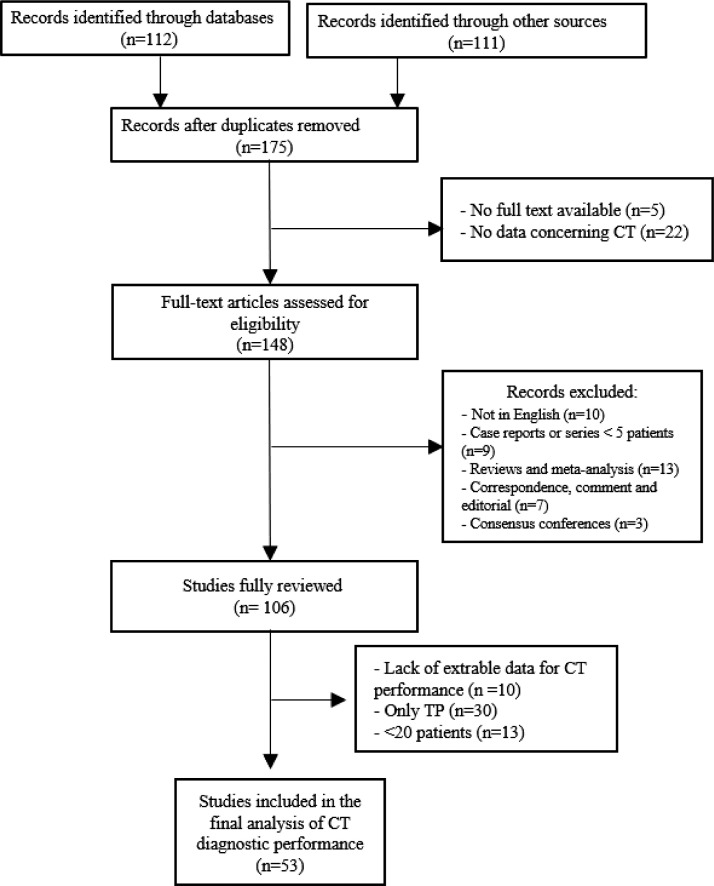
PRISMA flowchart.

Our literature search resulted in 175 unique studies after removal of duplicates. Records that included only an abstract (*n* = 5), were non-English and did not have extractable imaging data (*n* = 10), and those that had no imaging data at all (*n* = 22) were automatically excluded. We screened the remaining studies and removed case reports or series of less than 5 patients (*n* = 9), correspondences, comments, and editorials (*n* = 7), consensus conferences (*n* = 3), and reviews/meta-analyses (*n* = 13). In total, 106 publications fulfilled our search criteria and were fully reviewed. For our analysis of diagnostic accuracy, we further curated the studies under consideration. Of the 106 surveyed, 96 had extractable data for a two-by-two contingency table of diagnostic performance, but 30 of these were removed as they only reported the true-positive number of patients. Additionally, 13 studies that included fewer than 20 patients were removed to improve statistical significance, leaving 53 reports for our final analysis [3,5,6,8,[13], [14], [15], [16], [17], [18], [19], [20], [21], [22], [23], [24], [25], [26], [27], [28], [29], [30], [31], [32], [33], [34], [35], [36], [37], [38], [39], [40], [41], [42], [43], [44], [45], [46], [47], [48], [49], [50], [51], [52], [53], [54], [55], [56], [57], [58], [59], [60], [61]], (Supplementary table A). The PRISMA flowchart of literature search and study selection process is shown in Fig 1.

Among the publications identified for diagnostic accuracy evaluation, 92% (49/53) were retrospective studies, 4% (2/53) were case reports/case series with more than 5 patients, and only 4% (2/53) were prospective studies. Most of the reviewed works (*n* = 41/53, 77%) were monocentric and locally based. Moreover, only 23% (*n* = 12) were national multicenter studies and only one was multinational.

### 3.2 CT diagnostic performances

**Fig. 2:**
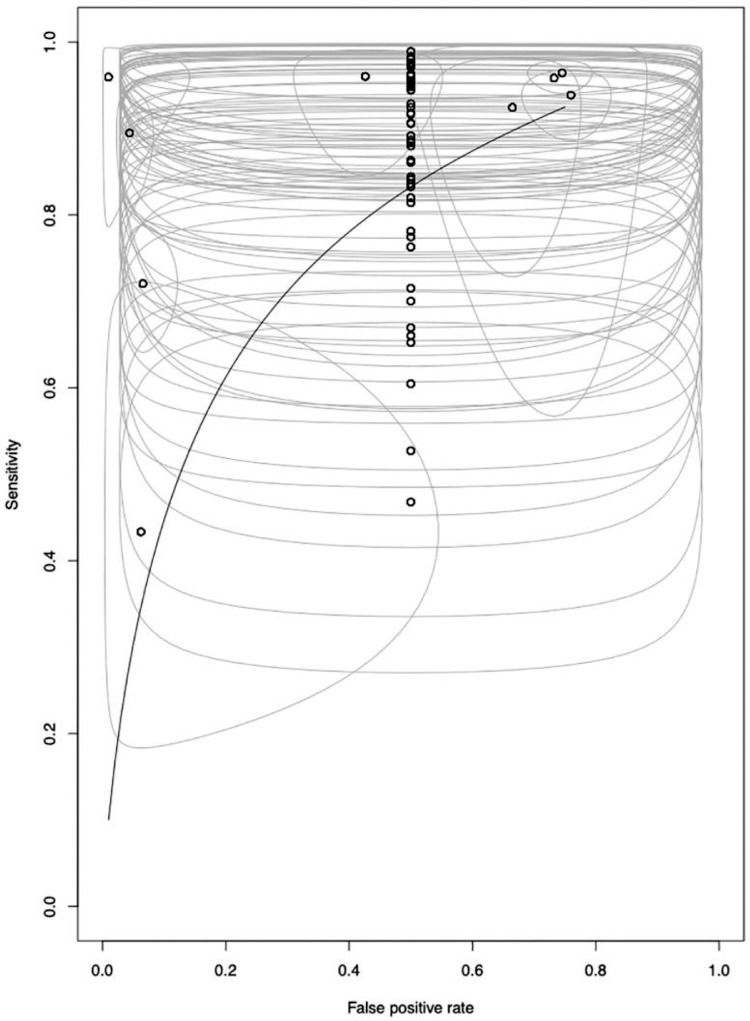
ROC curve of chest CT for diagnosis of COVID-19., The pooled performance was estimated (black line). The pooled false positive rate is displayed as a function of pooled sensitivity (black line). The FPR and Se for each study are displayed (dots) as well as their estimated 95 confidence intervals (gray line).

**Fig. 3:**
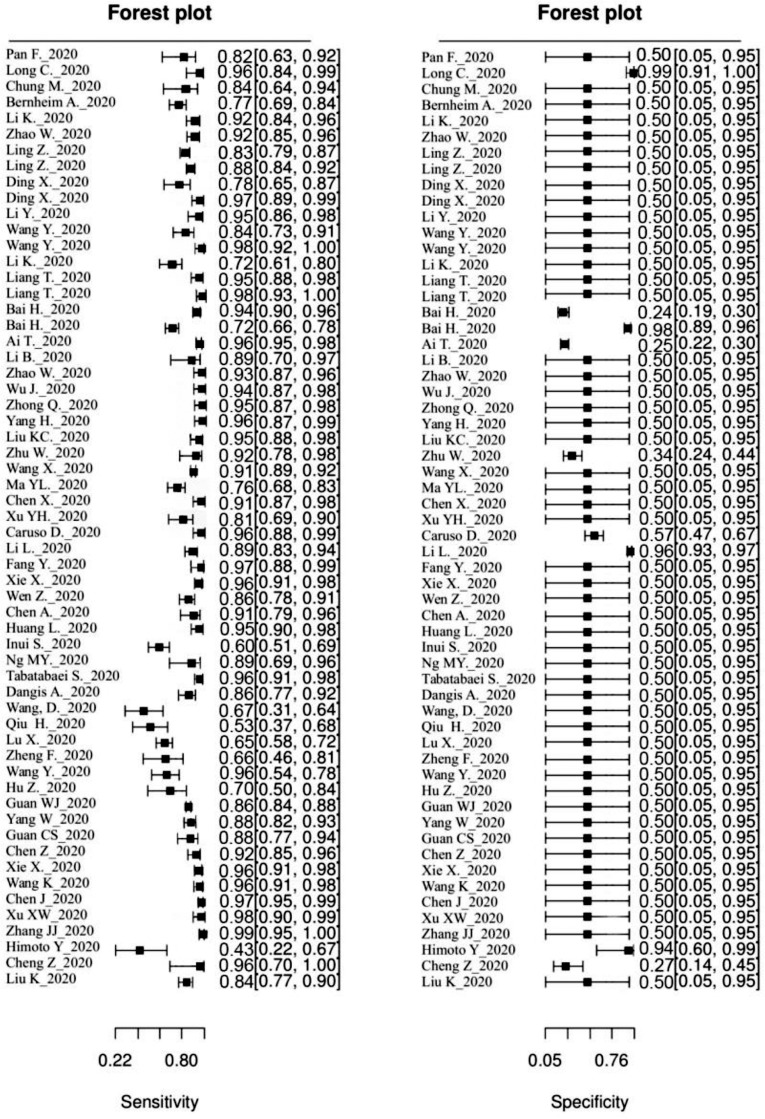
Forest plots of sensitivity and specificity of chest CT for the diagnosis of COVID-19., The Sensitivity and Specificity for each study is displayed as well as their estimated 95 confidence interval.

Of the 53 studies reviewed for diagnostic accuracy, 6 presented data on two different populations, allowing performance calculations to be carried out for each cohort individually. Overall, our analysis of chest CT sensitivity for the detection of COVID-19 pneumonia showed promising results: In 51% of cases (30/59), the sensitivity was found to be above 0.9, with varying confidence intervals (Fig 2). However, chest CT specificity remained low at just 0.565 (IC95 [0.539; 0.591]). In 93% of cases (55/59), a specificity lower than or equal to 0.5 was reported (Fig 3).

### 3.3 Oncologic population

Only 15 of the original 106 reports (14.2%) identified in our search discretely included cancer patients; 92 (86.8%) made no mention of cancer rates in their study populations and subsequently failed to demarcate these patients within the data, 1 study (0.9%) identified and excluded cancer patients while 13 (12.2%) indicated the presence of cancer in their cohorts but without further details. Finally, 3 studies (2.8%) pertained specifically to oncologic populations, but none were included in the final analysis due to only reporting true positives (*n* = 2) or outright lack of performance data (*n* = 1). Overall, only 7 studies that included and described cancer patients were included in the final analysis for chest CT performance. The aggregate prevalence of cancer across all studies surveyed was 0.3% (34/11,352 patients), with absolute numbers in individual reports ranging from 1 to 17 patients. Therefore, a specific analysis of chest CT performance in the oncologic population could not be done.

## 4 Discussion

In this systematic review and meta-analysis on chest CT imaging for the diagnosis of COVID-19 pneumonia, performed from 01/02/2020 to 04/23/2020 with a special focus on oncologic populations, our main finding was that there exists a dearth of research specifically addressing cancer patients. Among the 53 articles included in our performance analysis, sensitivity of chest CT was 0.889, with a low specificity of 0.565 (IC95 [0.539; 0.591]), falling below 0.5 in 90% of studies. Moreover, only 7 articles (7/53, 13%) specifically mentioned oncology patients in their results, rendering an evaluation of chest CT performance in this population impossible to conduct. The number of cancer patients in the individual studies included in our meta-analysis varied between 1 and 17 patients, for an aggregate total of 34 patients (0.3% of 11,352 patients included). Cancer prevalence in these cohorts was therefore much lower than the global figure of 5.8% reported in WHO statistics [62]. Among all reviewed studies, only one (1/106, 1%) specifically reported performance in the cancer patient group, but unfortunately only reported true positive patients, precluding further analysis. Our results are consistent with existing literature which has confirmed the high sensitivity of CT for the diagnosis of COVID-19, ranging from 89.76% (CI95 [84.42%; 93.84%]) to 94.6% (CI95 [91.9%; 96.45%]) [63,64]. More recent literature, published after completion of our study, shows improved values for specificity ranging from 87.2% (CI95 [83.9%; 89.9%]) to 91% (CI95 [91%; 92%]) [7,65]. Of note, these studies do not detail the prevalence of cancer in their study populations.

Several challenges regarding cancer management have arisen in the era of COVID-19. Primarily, although still poorly defined, the incidence of COVID-19 appears to be almost doubled in oncology populations when compared to the general population [66,67], with cancer patients facing a higher risk of severe disease manifestation requiring invasive airway management. This problem is compounded by the relatively older age of cancer patients, as age correlates with a higher frequency of hospitalization and severe disease [66,67], and the increased likelihood of COVID-19 exposure associated with frequent hospital visits for disease monitoring and treatment [68]. Moreover, the immunocompromising nature of both cancer and the associated treatment may make patients more susceptible to infections, though this point remains controversial as the estimated probability of death in infected patients with cancer ranges broadly from 13% to 33.1%[69,70]. Some studies have even reported similar death rates between cancer and non-cancer groups [71]. Finally, as a result of the pandemic, the quality of oncological care may suffer due to cancelation or postponement of non-urgent procedures and examinations, with possible consequences for patients who are lost to follow-up. According to a survey by the American Cancer Society Cancer Action Network (ACSCAN) during the first wave of COVID-19, 27% of patients undergoing active cancer treatment reported an interruption of a care, of which one out of five was related to a follow-up imaging exam [72]. In light of these findings, it is clear that the principle dilemma for oncologists has been the prioritization of tests and follow-up, balancing risk and benefit in the context of the ongoing pandemic [73]. Recommendations by national and international societies can shed light on some of these difficult situations [74], [75], [76], and radiologists in particular may emerge as leading figures in pandemic management for oncology patients. The latter must learn to expertly recognize COVID-19 pneumonia, meet the expectations of each clinical contact, particularly in the context of oncology, and prioritize examinations according to individual cases. They must also be familiar with all possible differential diagnoses relating to a clinical situation and must be cognizant of the possibility for incidentalomas [77]. Above all, they must reorganize and adapt to the new proposed care pathway and create reports within the current medical landscape defined by COVID-19. This last piece may prove to be the most challenging, considering the torrent of often contradictory recommendations being published at an ever-increasing pace by national and international bodies.

The role of chest CT for the initial management of COVID-19 patients without underlying cancer is currently well established, guided by relatively homogenous international recommendations [78]. Although most patients now arrive at the emergency room with a positive PCR, the usefulness of CT remains steadfast, allowing for correction of possible false negatives, assistance in the construction of differential diagnoses, and orientation of patients with prognostic information [79]. The current study suffers from a few limitations. First, the date range of our survey only covers the first wave of pandemic. Since this time, and as described above, the role of CT has slowly evolved due to the improved availability of antigenic and PCR testing. In addition, it would be useful to have more information available about the specific cancer cases described in the literature, including the date of diagnosis, whether or not it was active disease, and the treatment in progress. These additional pieces of data could be correlated with CT diagnostic performance. In conclusion, this meta-analysis highlights the lack of data concerning radiologic evaluation of COVID-19 cases in patients with underlying cancer. Further studies are required for this nuanced and highly sensitive population in order to better understand the specific performances of imaging techniques, and thereby lead to the improvement of disease management.

## CRediT authorship contribution statement

**Sylvain Bourdoncle:** Investigation, Writing – original draft, Resources. **Thomas Eche:** Writing – review & editing, Resources. **Jeremy McGale:** Writing – review & editing. **Kevin Yiu:** Writing – review & editing. **Ephraïm Partouche:** Methodology, Writing – review & editing. **Randy Yeh:** Writing – review & editing, Resources. **Samy Ammari:** Writing – review & editing, Resources. **Hervé Rousseau:** Validation. **Laurent Dercle:** Conceptualization, Methodology, Data curation, Software, Formal analysis, Writing – review & editing. **Fatima-Zohra Mokrane:** Conceptualization, Methodology, Supervision, Writing – review & editing.

## Declaration of Competing Interest

The authors declare that they have no known competing financial interests or personal relationships that could have appeared to influence the work reported in this paper.

## Appendix Supplementary materials

Image, application 1

## References

[1] « WHO Coronavirus (COVID-19) Dashboard ». https://covid19.who.int

[2] Lu X., Zhang L., Du H., Zhang J., Li Y.Y., Qu J. (2020). SARS-CoV-2 infection in children. N Engl J Med.

[3] Yang H., Sun G., Tang F., Peng M., Gao Y., Peng J. (2020). Clinical features and outcomes of pregnant women suspected of coronavirus disease 2019. J Infect.

[4] Desai A., Sachdeva S., Parekh T., Desal R. (2022). COVID-19 and Cancer: lessons from a pooled meta-analysis. JCO Glob Oncol.

[5] Inui S., Fujikawa A., Jitsu M., Kunishima N., Watanabe S., Suzuki Y. (2020 Mar 17). Chest CT Findings in Cases from the Cruise Ship “Diamond Princess” with Coronavirus Disease 2019 (COVID-19). Radiology.

[6] Zhang J.-.J., Dong X., Cao Y.-.Y., Yuan Y.-.D., Yang Y.-.B., Yan Y.-Q. (2020). Clinical characteristics of 140 patients infected with SARS-CoV-2 in Wuhan, China. Allergy.

[7] Herpe G., Lederlin M., Naudin M. (2021 Feb). Efficacy of Chest CT for COVID-19 Pneumonia Diagnosis in France. Radiology.

[8] Ai T., Yang Z., Hou H., Zhan C., Chen C., Lv W. (2020 Feb). Correlation of Chest CT and RT- PCR Testing in Coronavirus Disease 2019 (COVID-19) in China: a Report of 1014 Cases. Radiology.

[9] ACR Recommendations for the use of Chest Radiography and Computed Tomography (CT) for Suspected COVID-19 Infection. Available from: https://www.acr.org/Advocacy-and-Economics/ACR-Position-Statements/Recommendations-for-Chest-Radiography-and-CT-for-Suspected-COVID19-Infection.

[10] CDC. Coronavirus Disease 2019 (COVID-19) [Internet]. Centers for Disease Control and Prevention. 2020. https://www.cdc.gov/coronavirus/2019-ncov/hcp/testingoverview.html

[11] Radiology. COVID-19. Original research. https://pubs.rsna.org/2019-ncov_articles.

[12] European Radiology. Latest articles on COVID-19. https://www.european-radiology.org/highlights/covid-19.

[13] Pan F., Ye T., Sun P., Gui S., Liang B., Li L., Zheng D., Wang J., Hesketh R.L., Yang L., Zheng C. (2020 Jun). Time Course of Lung Changes at Chest CT during Recovery from Coronavirus Disease 2019 (COVID-19). Radiology.

[14] Long C., Xu H., Shen Q., Zhang X., Fan B., Wang C., Zeng B., Li Z., Li X., Li H. (2020 May). Diagnosis of the Coronavirus disease (COVID-19): rRT-PCR or CT?. Eur J Radiol.

[15] Chung M., Bernheim A., Mei X., Zhang N., Huang M., Zeng X., Cui J., Xu W., Yang Y., Fayad Z.A., Jacobi A., Li K., Li S., Shan H. (2020 Apr). CT imaging features of 2019 novel coronavirus (2019-nCoV). Radiology.

[16] Bernheim A., Mei X., Huang M., Yang Y., Fayad Z.A., Zhang N., Diao K., Lin B., Zhu X., Li K., Li S., Shan H., Jacobi A., Chung M. (2020 Jun). Chest CT findings in Coronavirus Disease-19 (COVID-19): relationship to duration of infection. Radiology.

[17] Li K., Wu J., Wu F., Guo D., Chen L., Fang Z., Li C. (2020 Jun). The clinical and chest CT features associated with severe and critical COVID-19 Pneumonia. Invest Radiol..

[18] Zhao W., Zhong Z., Xie X., Yu Q., Liu J. (2020 May). Relation between chest CT findings and clinical conditions of coronavirus disease (COVID-19) Pneumonia: a multicenter study. AJR Am J Roentgenol.

[19] Ling Z., Xu X., Gan Q., Zhang L., Luo L., Tang X., Liu J. (2020 May). Asymptomatic SARS-CoV-2 infected patients with persistent negative CT findings. Eur J Radiol.

[20] Ding X., Xu J., Zhou J., Long Q. (2020 Jun). Chest CT findings of COVID-19 pneumonia by duration of symptoms. Eur J Radiol.

[21] Li Y., Xia L. (2020 Jun). Coronavirus Disease 2019 (COVID-19): role of Chest CT in diagnosis and management. AJR Am J Roentgenol.

[22] Wang Y., Dong C., Hu Y., Li C., Ren Q., Zhang X., Shi H., Zhou M. (2020 Aug). Temporal changes of CT findings in 90 patients with COVID-19 Pneumonia: a longitudinal study. Radiology.

[23] Li K., Fang Y., Li W., Pan C., Qin P., Zhong Y., Liu X., Huang M., Liao Y., Li S. (2020 Aug). CT image visual quantitative evaluation and clinical classification of coronavirus disease (COVID-19). Eur Radiol.

[24] Liang T., Liu Z., Wu C.C., Jin C., Zhao H., Wang Y., Wang Z., Li F., Zhou J., Cai S., Liang Y., Zhou H., Wang X., Ren Z., Yang J. (2020 Sep). Evolution of CT findings in patients with mild COVID-19 pneumonia. Eur Radiol.

[25] Bai H.X. (2020). Performance of radiologists in differentiating COVID-19 from Non-COVID-19 Viral Pneumonia at chest CT. Radiology.

[26] Li B., Shen J., Li L., Yu C. (2020 May). Radiographic and clinical features of children with Coronavirus Disease (COVID-19) Pneumonia. Indian Pediatr.

[27] Zhao W., Zhong Z., Xie X., Yu Q., Liu J. (2020). CT Scans of Patients with 2019 Novel Coronavirus (COVID-19) Pneumonia. Theranostics.

[28] Wu J., Wu X., Zeng W., Guo D., Fang Z., Chen L., Huang H., Li C. (2020 May). Chest CT findings in patients with Coronavirus Disease 2019 and its relationship with clinical features. Invest Radiol.

[29] Zhong Q., Li Z., Shen X., Xu K., Shen Y., Fang Q., Chen F., Liang T. (2020 May 25). [CT imaging features of patients with different clinical types of coronavirus disease 2019 (COVID-19)]. Zhejiang Da Xue Xue Bao Yi Xue Ban. Chinese.

[30] Liu K.C., Xu P., Lv W.F., Qiu X.H., Yao J.L., Gu J.F., Wei W. (2020 May). CT manifestations of coronavirus disease-2019: a retrospective analysis of 73 cases by disease severity. Eur J Radiol.

[31] Zhu W., Xie K., Lu H., Xu L., Zhou S., Fang S. (2020 Sep). Initial clinical features of suspected coronavirus disease 2019 in two emergency departments outside of Hubei, China. J Med Virol.

[32] Wang X., Fang J., Zhu Y., Chen L., Ding F., Zhou R., Ge L., Wang F., Chen Q., Zhang Y., Zhao Q. (2020 Aug). Clinical characteristics of non-critically ill patients with novel coronavirus infection (COVID-19) in a Fangcang Hospital. Clin Microbiol Infect.

[33] Ma Y.L., Xia S.Y., Wang M., Zhang S.M., WH D.U., Chen Q. (2020 Apr). Clinical features of children with SARS-CoV-2 infection: an analysis of 115 cases. Zhongguo Dang Dai Er Ke Za Zhi. Chinese.

[34] Chen X., Tang Y., Mo Y., Li S., Lin D., Yang Z., Yang Z., Sun H., Qiu J., Liao Y., Xiao J., Chen X., Wu X., Wu R., Dai Z (2020 Sep). A diagnostic model for coronavirus disease 2019 (COVID-19) based on radiological semantic and clinical features: a multi-center study. Eur Radiol.

[35] Xu Y.H., Dong J.H., An W.M., Lv X.Y., Yin X.P., Zhang J.Z., Dong L., Ma X., Zhang H.J., Gao B.L. (2020 Apr). Clinical and computed tomographic imaging features of novel coronavirus pneumonia caused by SARS-CoV-2. J Infect.

[36] Caruso D., Zerunian M., Polici M., Pucciarelli F., Polidori T., Rucci C., Guido G., Bracci B., De Dominicis C., Laghi A. (2020 Aug). Chest CT features of COVID-19 in Rome, Italy. Radiology..

[37] Li L., Qin L., Xu Z., Yin Y., Wang X., Kong B., Bai J., Lu Y., Fang Z., Song Q., Cao K., Liu D., Wang G., Xu Q., Fang X., Zhang S., Xia J., Xia J. (2020 Aug). Using artificial intelligence to detect COVID-19 and community-acquired pneumonia based on pulmonary ct: evaluation of the diagnostic accuracy. Radiology.

[38] Fang Y., Zhang H., Xie J., Lin M., Ying L., Pang P., Ji W (2020 Aug). Sensitivity of Chest CT for COVID-19: comparison to RT-PCR. Radiology.

[39] Xie X., Zhong Z., Zhao W., Zheng C., Wang F., Liu J. (2020 Aug). Chest CT for typical Coronavirus Disease 2019 (COVID-19) Pneumonia: relationship to negative RT-PCR testing. Radiology.

[40] Wen Z., Chi Y., Zhang L., Liu H., Du K., Li Z., Chen J., Cheng L., Wang D. (2020 Apr 6). Coronavirus disease 2019: initial detection on chest CT in a retrospective multicenter study of 103 Chinese patients. Radiol Cardiothorac Imaging.

[41] Chen A., Huang J.X., Liao Y., Liu Z., Chen D., Yang C., Yang R.M., Wei X. (2020 Apr 6). Differences in clinical and imaging presentation of pediatric patients with COVID-19 in comparison with adults. Radiol Cardiothorac Imaging.

[42] Huang L., Han R., Ai T., Yu P., Kang H., Tao Q., Xia L (2020 Mar 30). Serial quantitative chest CT assessment of COVID-19: a deep learning approach. Radiol Cardiothorac Imaging.

[43] Ng M.Y., Lee E.Y.P., Yang J., Yang F., Li X., Wang H., Lui M.M., Lo C.S., Leung B., Khong P.L., Hui C.K., Yuen K.Y., Kuo M.D. (2020 Feb 13). Imaging Profile of the COVID-19 Infection: radiologic Findings and Literature Review. Radiol Cardiothorac Imaging.

[44] Tabatabaei S.M.H., Talari H., Moghaddas F., Rajebi H. (2020 Apr 20). CT features and short-term prognosis of COVID-19 Pneumonia: a single-center study from Kashan, Iran. Radiol Cardiothorac Imaging.

[45] Dangis A., Gieraerts C., De Bruecker Y., Janssen L., Valgaeren H., Obbels D., Gillis M., Van Ranst M., Frans J., Demeyere A., Symons R. (2020 Apr 21). Accuracy and reproducibility of low-dose submillisievert chest CT for the diagnosis of COVID-19. Radiol Cardiothorac Imaging.

[46] Wang D., Ju X.L., Xie F., Lu Y., Li F.Y., Huang H.H., Fang X.L., Li Y.J., Wang J.Y., Yi B., Yue J.X., Wang J., Wang L.X., Li B., Wang Y., Qiu B.P., Zhou Z.Y., Li K.L., Sun J.H., Liu X.G., Li G.D., Wang Y.J., Cao A.H., Chen Y.N. (2020 Apr 2). Clinical analysis of 31 cases of 2019 novel coronavirus infection in children from six provinces (autonomous region) of northern China. Zhonghua Er Ke Za Zhi. Chinese.

[47] Qiu H., Wu J., Hong L., Luo Y., Song Q., Chen D. (2020 Jun). Clinical and epidemiological features of 36 children with coronavirus disease 2019 (COVID-19) in Zhejiang, China: an observational cohort study. Lancet Infect Dis.

[48] Lu X., Zhang L., Du H., Zhang J., Li Y.Y., Qu J., Zhang W., Wang Y., Bao S., Li Y., Wu C., Liu H., Liu D., Shao J., Peng X., Yang Y., Liu Z., Xiang Y., Zhang F., Silva R.M., Pinkerton K.E., Shen K., Xiao H., Xu S., Wong G.W.K. (2020 Apr 23). Chinese pediatric novel coronavirus study team. SARS-CoV-2 infection in children. N Engl J Med.

[49] Zheng F., Liao C., Fan Q.H., Chen H.B., Zhao X.G., Xie Z.G., Li X.L., Chen C.X., Lu X.X., Liu Z.S., Lu W., Chen C.B., Jiao R., Zhang A.M., Wang J.T., Ding X.W., Zeng Y.G., Cheng L.P., Huang Q.F., Wu J., Luo X.C., Wang Z.J., Zhong Y.Y., Bai Y., Wu X.Y., Jin R.M. (2020 Apr). Clinical characteristics of children with coronavirus disease 2019 in Hubei, China. Curr Med Sci.

[50] Wang Y., Liu Y., Liu L., Wang X., Luo N., Li L. (2020 May 11). Clinical outcomes in 55 patients with severe acute respiratory syndrome coronavirus 2 who were asymptomatic at hospital admission in Shenzhen, China. J Infect Dis..

[51] Hu Z., Song C., Xu C., Jin G., Chen Y., Xu X., Ma H., Chen W., Lin Y., Zheng Y., Wang J., Hu Z., Yi Y., Shen H (2020 May). Clinical characteristics of 24 asymptomatic infections with COVID-19 screened among close contacts in Nanjing, China. Sci China Life Sci..

[52] Guan W.J., Ni Z.Y., Hu Y., Liang W.H., Ou C.Q., He J.X., Liu L., Shan H., Lei C.L., Hui D.S.C., Du B., Li L.J., Zeng G., Yuen K.Y., Chen R.C., Tang C.L., Wang T., Chen P.Y., Xiang J., Li S.Y., Wang J.L., Liang Z.J., Peng Y.X., Wei L., Liu Y., Hu Y.H., Peng P., Wang J.M., Liu J.Y., Chen Z., Li G., Zheng Z.J., Qiu S.Q., Luo J., Ye C.J., Zhu S.Y., Zhong N.S. (2020 Apr 30). China Medical Treatment Expert Group for Covid-19. Clinical Characteristics of Coronavirus Disease 2019 in China. N Engl J Med.

[53] Yang W. (2020 Apr). Clinical characteristics and imaging manifestations of the 2019 novel coronavirus disease (COVID-19):a multi-center study in Wenzhou city, Zhejiang, China. J Infect.

[54] Guan C.S., Lv Z.B., Yan S., Du Y.N., Chen H., Wei L.G., Xie R.M., Chen B.D. (2020 May). Imaging features of coronavirus disease 2019 (COVID-19): evaluation on thin-section CT. Acad Radiol.

[55] Chen Z., Fan H., Cai J., Li Y., Wu B., Hou Y., Xu S., Zhou F., Liu Y., Xuan W., Hu H., Sun J. (2020 May). High-resolution computed tomography manifestations of COVID-19 infections in patients of different ages. Eur J Radiol.

[56] Wang K., Kang S., Tian R., Zhang X., Zhang X., Wang Y. (2020 May). Imaging manifestations and diagnostic value of chest CT of coronavirus disease 2019 (COVID-19) in the Xiaogan area. Clin Radiol.

[57] Chen J., Qi T., Liu L., Ling Y., Qian Z., Li T., Li F., Xu Q., Zhang Y., Xu S., Song Z., Zeng Y., Shen Y., Shi Y., Zhu T., Lu H. (2020 May). Clinical progression of patients with COVID-19 in Shanghai, China. J Infect..

[58] Xu X.W., Wu X.X., Jiang X.G., Xu K.J., Ying L.J., Ma C.L., Li S.B., Wang H.Y., Zhang S., Gao H.N., Sheng J.F., Cai H.L., Qiu Y.Q., Li L.J. (2020 Feb 19). Clinical findings in a group of patients infected with the 2019 novel coronavirus (SARS-Cov-2) outside of Wuhan, China: retrospective case series. BMJ.

[59] Himoto Y., Sakata A., Kirita M., Hiroi T., Kobayashi K.I., Kubo K., Kim H., Nishimoto A., Maeda C., Kawamura A., Komiya N., Umeoka S. (2020 May). Diagnostic performance of chest CT to differentiate COVID-19 pneumonia in non-high-epidemic area in Japan. Jpn J Radiol.

[60] Cheng Z., Lu Y., Cao Q., Qin L., Pan Z., Yan F., Yang W. (2020 Jul). Clinical features and chest CT manifestations of Coronavirus Disease 2019 (COVID-19) in a single-center study in Shanghai, China. AJR Am J Roentgenol.

[61] Liu K., Fang Y.Y., Deng Y., Liu W., Wang M.F., Ma J.P., Xiao W., Wang Y.N., Zhong M.H., Li C.H., Li G.C., Liu H.G (2020 May 5). Clinical characteristics of novel coronavirus cases in tertiary hospitals in Hubei Province. Chin Med J (Engl).

[62] World Health Organization. Cancer Today. 2020. http://gco.iarc.fr/today/home.

[63] Adams H.J.A., Kwee T.C., Yakar D., Hope M.D., Kwee R.M. (2022). Systematic review and meta-analysis on the value of chest CT in the diagnosis of Coronavirus Disease (COVID-19): sol scientiae, illustra Nos. Am J Roentgenol.

[64] Bao C., Liu X., Zhang H., BSC, Li Y., Liu J. (2020 Jun; 17). Coronavirus Disease 2019 (COVID-19) CT Findings: a systematic review and meta-analysis. J Am Coll Radiol.

[65] Lieveld A.W.E., Azijli K., Teunissen B.P., van Haaften R.M., Kootte R.S., van den Berk I.A.H., van der Horst S.F.B., de Gans C., van de Ven P.M., Nanayakkara P.W.B. (2021 Mar). Chest CT in COVID-19 at the ED: validation of the COVID-19 Reporting and Data System (CO-RADS) and CT severity score: a prospective, multicenter, observational study. Chest.

[66] Liang W., Guan W., Chen R., Wang W., Li J., Xu K. (2020). Cancer patients in SARS-CoV-2 infection: a nationwide analysis in China. Lancet Oncol.

[67] Zhang L., Zhu F., Xie L., Wang C., Wang J., Chen R. (2020). Clinical characteristics of COVID-19-infected cancer patients: a retrospective case study in three hospitals within Wuhan, China. Ann Oncol.

[68] Yu J., Ouyang W., Chua M.L.K., Xie C. (2020). SARS-CoV-2 transmission in patients with cancer at a tertiary care hospital in Wuhan, China. JAMA Oncol.

[69] Kuderer N.M., Choueiri T.K., Shah D.P., Shyr Y., Rubinstein S.M., Rivera D.R., Shete S., Hsu C.Y., Desai A., de Lima Lopes G., Grivas P., Painter C.A., Peters S., Thompson M.A., Bakouny Z., Batist G., Bekaii-Saab T., Bilen M.A., Bouganim N., Larroya M.B., Castellano D., Del Prete S.A., Doroshow D.B., Egan P.C., Elkrief A., Farmakiotis D., Flora D., Galsky M.D., Glover M.J., Griffiths E.A., Gulati A.P., Gupta S., Hafez N., Halfdanarson T.R., Hawley J.E., Hsu E., Kasi A., Khaki A.R., Lemmon C.A., Lewis C., Logan B., Masters T., McKay R.R., Mesa R.A., Morgans A.K., Mulcahy M.F., Panagiotou O.A., Peddi P., Pennell N.A., Reynolds K., Rosen L.R., Rosovsky R., Salazar M., Schmidt A., Shah S.A., Shaya J.A., Steinharter J., Stockerl-Goldstein K.E., Subbiah S., Vinh D.C., Wehbe F.H., Weissmann L.B., Wu J.T., Wulff-Burchfield E., Xie Z., Yeh A., Yu P.P., Zhou A.Y., Zubiri L., Mishra S., Lyman G.H., Rini B.I., Warner J.L. (2020 Jun 20). COVID-19 and Cancer Consortium. Clinical impact of COVID-19 on patients with cancer (CCC19): a cohort study. Lancet.

[70] de Melo A.C., Thuler L.C.S., da Silva J.L. (2020). Cancer inpatients with COVID-19: a report from the Brazilian National Cancer Institute. PLoS ONE.

[71] Aboueshia M., Hussein M.H., Attia A.S. (2021). Cancer and COVID-19: analysis of patient outcomes. Future Oncol.

[72] COVID-19 Pandemic Early Effects on Cancer Patients and Survivors: April 2020. American Cancer Society Cancer Action Network. 2020. https://www.fightcancer.org/policy-resources/covid-19-pandemic-early-effects-cancer-patients-and-survivors-april-2020.

[73] Hanna T.P., Evans G.A., Booth C.M. (2020). Cancer, COVID-19 and the precautionary principle: prioritizing treatment during a global pandemic. Nat Rev Clin Oncol.

[74] Tagliaferri L., Di Stefani A., Schinzari G., Fionda B., Rossi E., Del Regno L. (2020 Jun). Skin cancer triage and management during COVID-19 pandemic. J Eur Acad Dermatol Venereol.

[75] Schultz P., Morvan J.-B., Fakhry N., Morinière S., Vergez S., Lacroix C. (2020 May). French consensus regarding precautions during tracheostomy and post-tracheostomy care in the context of COVID-19 pandemic. Eur Ann Otorhinolaryngol Head Neck Dis.

[76] Akladios C., Azais H., Ballester M., Bendifallah S., Bolze P.-.A., Bourdel N. (2020 Jun). Recommendations for the surgical management of gynecological cancers during the COVID-19 pandemic - FRANCOGYN group for the CNGOF. J Gynecol Obstet Hum Reprod.

[77] Czernin J., Fanti S., Meyer P.T., Allen-Auerbach M., Hacker M., Sathekge M. (2020). Nuclear Medicine Operations in the Times of COVID-19: strategies, Precautions, and Experiences. J Nucl Med.

[78] Rubin G.D., Ryerson C.J., Haramati L.B., Sverzellati N., Kanne J.P., Raoof S., Schluger N.W., Volpi A., Yim J., Martin I.B.K., Anderson D.J., Kong C., Altes T., Bush A., Desai S.R., Goldin J., Goo J., Humbert Y., Inoue H., Kauczor F., Luo P.J., Mazzone M., Prokop M., Remy-Jardin L., Richeldi C.M., Schaefer-Prokop M., Tomiyama N., Wells A.U., Leung A.N. (2020). The role of chest imaging in patient management during the COVID-19 pandemic: a multinational consensus statement from the fleischner society. Radiology.

[79] Société Française de Radiologie. (2021). https://ebulletin.radiologie.fr/actualites-covid-19/actualisation-recommandations-dimagerie-thoracique-pneumonie-covid-19.

