# Supplementary material for: Investigating of the role of CT scan for cancer patients during the first wave of COVID-19 pandemic
Source: Res Diagn Interv Imaging. 2022 Mar 31;1:100004. doi: 10.1016/j.redii.2022.100004 (PMC8970534; doi:10.1016/j.redii.2022.100004)
Supplement: Supplementary file 1 [file mmc1.docx]

**Supplementary material**

| **Author** | **Date** | **Reference (PMID/DOI)** |
| --- | --- | --- |
| Pan F. | 13-Feb 2020 | 32053470 |
| Long C. | 25-Mar 2020 | 32229322 |
| Chung M. | 2-Apr 2020 | 32017661 |
| Bernheim A. | 20-Feb 2020 | 32077789 |
| Li K. | 29-Feb 2020 | 32118615 |
| Zhao W. | 3-Mar 2020 | 32125873 |
| Ling Z. | 12- Mar 2020 | 32199142 |
| Ding X. | 18-Apr 2020 | 32325282 |
| Li Y. | 3-Apr 2020 | 32130038 |
| Wang Y. | 6-Apr 2020 | 32191587 |
| Li K. | 25-Mar 2020 | 32215691 |
| Liang T. | 15-Apr 2020 | 32291502 |
| Bai H. | 10-Mar, 2020 | 32155105 |
| Ai T. | 26-Feb 2020 | 32101510 |
| Li B. | 7-Apr 2020 | 32255437 |
| Zhao W. | 15-Mar 2020 | 32292517 |
| Wu J. | 27-Feb 2020 | 32091414 |
| Zhong Q. | 25-Mar 2020 | 32207591 |
| Yang H. | 12-Apr 2020 | 32294503 |
| Liu KC. | 12-Mar 2020 | 32193037 |
| Zhu W. | 13-Mar 2020 | 32167181 |
| Wang X. | 3-Apr 2020 | 32251842 |
| Ma YL. | Apr, 2020 | 32312363 |
| Chen X. | 16-Apr 2020 | 32300971 |
| Xu YH. | 25-Feb 2020 | 32109443 |
| Caruso D. | 3-Apr 2020 | 32243238 |
| Li L. | 19-Mar 2020 | 32191588 |
| Fang Y. | 19-Feb 2020 | 32073353 |
| Xie X. | 12-Feb 2020 | 32049601 |
| Wen Z. | 6-Apr 2020 | 10.1148/ryct.2020200092 |
| Chen A. | 6-Apr, 2020 | 10.1148/ryct.2020200117 |
| Huang L. | 30-Mar 2020 | 10.1148/ryct.2020200075 |
| Inui S. | 17-Mar 2020 | 10.1148/ryct.2020200110 |
| Ng MY. | 13-Feb 2020 | 10.1148/ryct.2020200034 |
| Tabatabaei SMH. | 20-Apr 2020 | 10.1148/ryct.2020200130 |
| Dangis A. | 21-Apr 2020 | 10.1148/ryct.2020200196 |
| Wang D. | 7-Feb 2020 | 32118389 |
| Qiu  H. | 25-Mar 2020 | 32220650 |
| Lu X. | 23-Apr 2020 | 32187458 |
| Zheng F. | 24-Mar 2020 | 32207032 |
| Wang Y. | 17-Mar 2020 | 32179910 |
| Hu Z. | 4-Mar 2020 | 32146694 |
| Guan WJ. | 28-Feb 2020 | 32109013 |
| Yang W. | 26-Feb 2020 | 32112884 |
| Guan CS. | 20-Mar 2020 | 32204990 |
| Chen Z. | 24-Mar 2020 | 32240913 |
| Wang K. | 23-Mar 2020 | 32216961 |
| Chen J. | 19-Mar 2020 | 32171869 |
| Xu XW. | 19-Feb 2020 | 32075786 |
| Zhang JJ. | 19-Feb 2020 | 32077115 |
| Himoto Y. | 30-Mar 2020 | 32232648 |
| Cheng Z. | 14-Mar 2020 | 32174128 |
| Liu K. | 7-Feb 2020 | 32044814 |

**Supplemental Table A.** Exhaustive list of included articles for C-CT diagnosis performance analysis used in this metanalysis.
